# Electrodecoration and Characterization of Superparamagnetic Iron Oxide Nanoparticles with Bioactive Synergistic Nanocopper: Magnetic Hyperthermia-Induced Ionic Release for Anti-Biofilm Action

**DOI:** 10.3390/antibiotics10020119

**Published:** 2021-01-27

**Authors:** Verdiana Marchianò, Maria Salvador, Amanda Moyano, Gemma Gutiérrez, María Matos, Susana Yáñez-Vilar, Yolanda Piñeiro, José Rivas, José C. Martínez-García, Davide Peddis, Maria C. Blanco-López, Montserrat Rivas, Nicoletta Ditaranto, Nicola Cioffi

**Affiliations:** 1 Department of Physical and Analytical Chemistry, University of Oviedo, 33006 Oviedo, Spain; marchianoverdiana@uniovi.es (V.M.); moyanoamanda@uniovi.es (A.M.); cblanco@uniovi.es (M.C.B.-L.); 2 Department of Physics and IUTA, University of Oviedo, 33204 Gijon, Spain; salvadormaria@uniovi.es (M.S.); jcmg@uniovi.es (J.C.M.-G.); rivas@uniovi.es (M.R.); 3 Istituto di Struttura della Materia—Consiglio Nazionale delle Ricerche (CNR), Monterotondo Scalo (RM), 00016 Rome, Italy; davide.peddis@cnr.it; 4 Department of Chemical Engineering, University of Oviedo, 33006 Oviedo, Spain; gutierrezgemma@uniovi.es (G.G.); matosmaria@uniovi.es (M.M.); 5 Instituto Universitario de Biotecnología de Asturias, University of Oviedo, 33006 Oviedo, Spain; 6 Department of Applied Physics, University of Santiago de Compostela, 15782 Santiago de Compostela, Spain; susana.yanez@usc.es (S.Y.-V.); y.pineiro.redondo@usc.es (Y.P.); jose.rivas@usc.es (J.R.); 7 Dipartimento di Chimica and CSGI—Bari Unit, Università degli Studi di Bari Aldo Moro, 70125 Bari, Italy; nicola.cioffi@uniba.it

**Keywords:** copper nanoparticle, SPION, synergistic bioactivity, hyperthermia, ion release

## Abstract

The urgency for the availability of new antibacterial/disinfectant agents has become a worldwide priority. At the same time, along with the extensive use of other metal nanoparticles (NPs), the investigation of magnetic NPs (MNPs) in antibacterial studies has turned out to be an increasingly attractive research field. In this context, we present the preparation and characterization of superparamagnetic iron oxide NPs, electrodecorated with antimicrobial copper NPs, able to modulate the release of bioactive species not only by the NP’s stabilizer, but also through the application of a suitable magnetic field. Antimicrobial synergistic CuNPs stabilized by benzalkonium chloride have been used in the current study. We demonstrate the successful preparation of Cu@Fe_3_O_4_ MNPs composites through morphological and spectroscopic results. Additionally, an extensive magnetic characterization is reported, along with hyperthermia-induced copper ionic release. On the basis of our results, we propose a new generation of antimicrobial magnetic nanomaterials, whose bioactivity can be also tuned by the application of a magnetic field.

## 1. Introduction

Iron oxides are widespread compounds, which are well-known in nature and can be synthesized by low-cost scalable routes [1,2,3,4]. Superparamagnetic iron oxides (SPIONs) have many uses, standing out for bio-applications such as contrast agents for magnetic resonance imaging (MRI), targeted drug delivery, magnetic hyperthermia and thermoablation, bioseparation, and biosensing [5,6,7,8]. Eight iron oxides are known, among these, hematite (α-Fe_2_O_3_), magnetite (Fe_3_O_4_) and maghemite (γ-Fe_2_O_3_) have unique biochemical, magnetic, catalytic, and other properties which make them suitable for specific technical and biomedical applications. Magnetite is a black iron mixed oxide Fe (II)/Fe (III), which has different chemical–physical characteristics due to its oxidation state. The presence of Fe^2+^ allows it to act as an electron donor. Fe_3_O_4_ has a face centered cubic spinel structure based on 32 O^2−^ ions and is close-packed along all directions; it also has a cubic inverse spinel structure that consists of a cubic close packed array of oxide ions. In stoichiometric magnetite Fe^2+^/Fe^3+^ = 1/2, and the divalent irons may be partly or fully replaced by other divalent ions [8].

The SPIONs can be used for various bio-applications thanks to the correlation between their magnetic properties and their size and shape. Bulk magnetite is typically a ferrimagnetic material but when its size is reduced below a threshold (~15 nm) it has a superparamagnetic behavior at room temperature. The latter means that the thermal energy enables the magnetization of the particle to switch spontaneously between the two easy-magnetization directions with a certain relaxation time. As a consequence, the apparent magnetization of the particle is, in the absence of an applied magnetic field, nullified. On the other hand, when an exciting field is applied, the particle magnetizes and has a large saturation magnetization. In spite of their null remanent magnetization, the magnetite nanoparticles have a high specific surface area that makes the Van der Waals interactions significant. This can lead to clustering and the formation of large and small aggregates, with a smaller specific surface area [9,10,11]. Hence, for protecting bare SPIONs against aggregation, the magnetic properties can be tailored by the coating materials, such as Au, Ag and Co_3_O_4_. Magnetite can be synthesized by different processes, like the thermal decomposition, microemulsion, hydrothermal and co-precipitation methods [8]. The problem associated with magnetic iron oxide NPs (IONPs) in the size range is their intrinsic instability over longer periods, which manifests in two main ways: loss of dispersibility, where small nanoparticles (NPs) tend to aggregate and form large particles to reduce the surface energy, and loss of magnetism, where bare SPIONs are easily oxidized in air due to their high chemical activity, especially Fe_3_O_4_ and γ-Fe_2_O_3_ NPs. Therefore, it is crucial to develop a proper protection strategy to chemically stabilize bare SPIONs against damage during or after their subsequent application. For biomedical applications, it is necessary to obtain water dispersible NPs, because most biological media are nearly neutral aqueous solutions. Different types of nanoparticles structures have been designed: the core–shell structure, matrix dispersed structure, Janus-type heterostructures and shell–core–shell structure [8]. The biocompatibility and toxicity of SPIONs are important criteria to take into account for their biomedical applications. Parameters determining biocompatibility and toxicity are the nature of the magnetically responsive component, and the final size of the composite particles including their core and the coatings (shell). Ideally, composite SPIONs must also have a high magnetization, so that their movement in the blood can be controlled with an external magnetic field until they are immobilized close to the targeted pathologic tissue. SPIONs can cross cellular barriers, blood circulation, and penetrate into various organs. They are easily metabolized by the liver and eliminated by filtration from the kidney. Another advantage is that when the external magnetic field is no longer applied, they return to having zero magnetization. In the medical field, thanks to their magnetic properties and the ability to operate at the cellular level, SPIONs are used for therapeutic purposes such as drug delivery. Nanoparticles have been also been applied with an external magnet on the affected area—the target—reducing side and toxic effects in the surrounding areas [4,12,13]. In the diagnostic field, they are used for imaging and magnetic resonance [5,11,14,15,16,17,18,19].

One of the most outstanding applications of SPIONs is magnetic hyperthermia, which has been successfully tested to destroy tumor cells when combined with chemotherapy and radiotherapy [5]. Magnetic hyperthermia uses AC stimulation (radio-frequency range between kHz and 1 MHz) of magnetic NPs (MNPs) to produce a localized heating effect. Heating above 42 °C can selectively damage cancerous cells without harming the surrounding healthy tissue [8,14,20]. The size of the nanoparticles is extremely important for such use, because if too large, they cannot cross the barrier of endothelial cells to pass from the bloodstream to the target cell. At the same time, they must not be too small, because then the heat released by hyperthermia is not sufficient to cause the tumor’s death. These are the two fundamental characteristics for the application of hyperthermia [5].

There are other strategies for applying magnetic nanoparticles in chemotherapy such as incorporating antitumoral drugs, e.g., paclitaxel and doxorubicin, or functionalizing the surface with polymeric coating agents and biomolecules that help achieve the target [12,13]. Recent studies have evaluated the possibility of doping iron oxide nanoparticles with transition metal cations, optimizing the magnetic characteristics of iron oxide: anisotropy and magnetic moment. Cobalt was used to increase anisotropy, while zinc is used to decrease the magnetic moment [14]. The viability and cellular activity are measured in mesenchymal stem cells derived from primary human bone marrow and in cells derived from human osteosarcoma. The doping-induced changes in the magnetic response of nanoparticles, both as stable aqueous suspensions and when associated with cells, has been studied. The results show that the particles’ magnetic response was altered after cellular interaction with a reduction in their mobility. For both types of particles, it was found that the moderate doping levels required for optimal magnetic properties did not alter their cytotoxicity or influence the osteogenic differentiation of stem cells. Therefore, despite the known cytotoxicity of cobalt and zinc ions, these results suggest that iron oxide nanoparticles can be doped to sufficiently adapt their magnetic properties without compromising cell biocompatibility.

Very recently, the action of iron oxide has been tested as an antimicrobial, both alone [4,21,22] and in combination with other metal/metal oxides with well-known antimicrobial activity to exploit a synergistic effect [23,24,25,26,27]. Among them, the phenomenon of photo catalysis in bacterial inactivation under solar or LED irradiation has been deeply investigated in the last years: titanium oxide nanotubes incorporated into silver nanoparticles [11,28], as well as different IONPs composites with zinc, silver, and gold NPs [29,30,31,32]. 

In this background, the aim of this study is to explore a new usage of magnetic hyperthermia, not to destroy tumor cells as is traditional in novel cancer therapy, but for antimicrobial purposes. The combination of the well-known bactericidal copper nanoparticles (CuNPs) possessing controlled release properties with magnetic Fe_3_O_4_, gives rise to a composite material whose antimicrobial activity can be tuned through magnetic activation.

It is well known that the bioactivity of CuNPs can be easily tuned by modifying their loading (weight percentage) in the antimicrobial coating [33], or by changing the thickness of the NP stabilizing shell [34]. Both of these approaches allow one to tune the release of cupric ions, which has been demonstrated to be tightly correlated with the antibacterial properties of copper-based nanomaterials [33,34,35]. Hence, controlling the ionic release affords for tuning the final bioactivity of the materials. In this work, we present a third and orthogonal way to tune the ionic release and promote the bioactivity of CuNPs, by magnetic field control, thus providing an additional tool to gain the ultimate control over nanoantimicrobial efficiency.

## 2. Results and Discussion

### 2.1. Iron Oxide Preparation and Electrodecoration with Copper Nanoparticles

**Figure 1:**
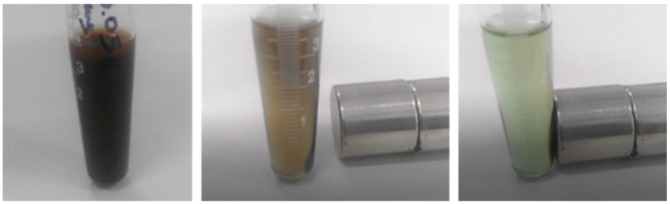
Magnetic character of Cu electrodecorated iron oxide colloidal suspension.

Different iron oxide nanoparticles were prepared by co-precipitation (see Section 3 for details), both capped and un-capped. The latter were obtained using two different polymers as capping agents, namely polyacrylic acid (PAA) and polyetherimide (PEI). Both polymer capped and un-stabilized SPIONs were used in the electrodecoration process, performed through the SAE technique [33,35,36,37]. The magnetite nanomaterials were directly added and dispersed in the electrochemical cell, along with the reaction solution containing the cationic surfactant benzyl-dimethyl-hexadecyl-ammonium chloride (BDHAC), which acted as the electrolyte and NPs stabilizer. In all the cases, the resulting colloid suspension demonstrated magnetic behavior which allowed the magnetic separation of the particles from the medium (Figure 1). Despite this behavior, the polymer capped SPIONs did not show any iron-copper interaction due to presence of both polymer and CuNPs stabilizer. Therefore, all the investigation was performed using only the un-stabilized SPIONs.

### 2.2. Morphological and Spectroscopic Characterization

**Figure 2:**
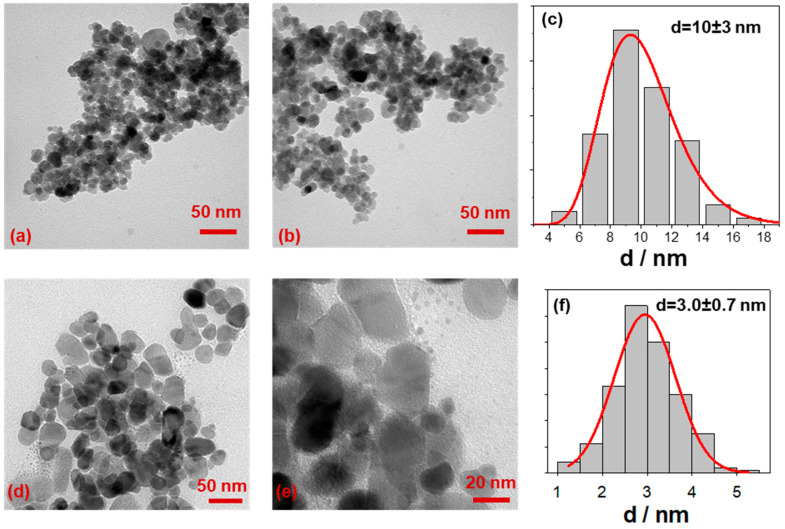
TEM pictures of (**a**,**b**) bare Fe_3_O_4_ magnetic nanoparticles (MNPs) and (**d**,**e**) of Cu@Fe_3_O_4_ MNPs. Size distribution histograms of (**c**) bare MNPs and (**f**) supported CuNPs.

The SPIONs morphology is represented in Figure 2, where transmission electron microscopy (TEM) images of bare and electrodecorated Fe_3_O_4_ are reported, along with the relative size distribution histograms. As-prepared magnetite is characterized by a “grape structure” morphology with spherical shaped particles, as evident in Figure 2a,b. The average size diameter has been measured to be 10 ± 3 nm (Figure 2c).

After the electrodecoration process, the magnetite morphology is preserved. In addition the presence of small particles—CuNPs—close to/onto the grape structure of iron oxide NPs, with a mean size diameter of 3.0 ± 0.7 nm can be seen (Figure 2c–e). This good result has been obtained acting on the applied voltage and on the amount of CuNPs stabilizer during the electrochemical preparation. Mainly, the proper BDHAC concentration was deeply investigated to stabilize the forming CuNPs without preventing the interaction between copper clusters and iron oxide nanoparticles. 

SPIONs surface was analyzed by x-ray photoelectron spectroscopy (XPS) to study their surface chemical composition and to investigate how this is affected after the electrodecoration with copper. Elemental surface composition is reported in Table 1.

After the electrodecoration, a significant amount of copper was found on the surface of the SPIONs, along with nitrogen, chlorine detection and carbon increases, all coming from the BDHAC molecules used as the electrolyte and surfactant during the electrolysis procedure. Nevertheless, iron is still exposed and revealed in the copper modified magnetite. Table 1 also reports inorganic oxygen (O-oxide) relative abundance, as calculated after O1s signal curve fitting (spectra not reported). This value was related to Fe% and the O/Fe ratio was calculated. A value close to 2 was found both for pristine and electrodecorated SPIONs, compatible with α-/γ –FeOOH and α-/γ –Fe_2_O_3_ [38], indicating that no further oxidation occurred during the electrodecoration. 

**Figure 3:**
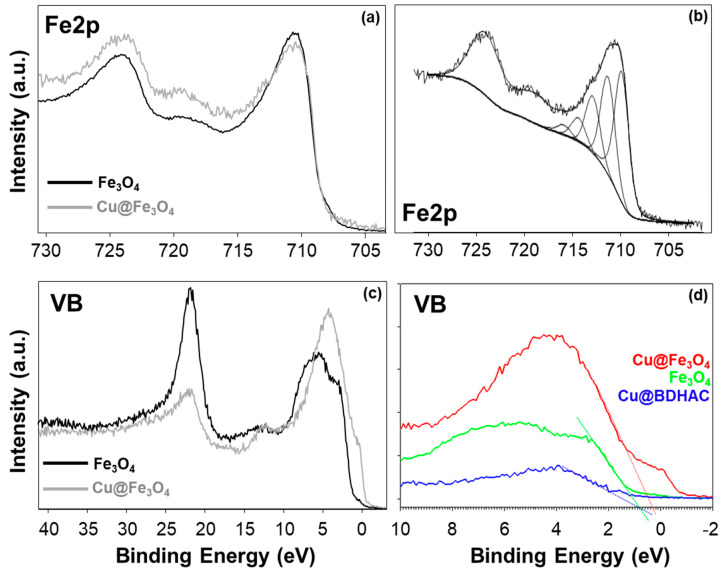
XP spectra of Fe_3_O_4_ and Cu@Fe_3_O_4_ nanomaterials. (**a**) Fe2p spectra comparison; (**b**) Fe2p typical curve fitting; (**c**) valence band comparison; (**d**) Fermi level extrapolation from valence band spectra.

XPS characterization of magnetite nanomaterials has been described to be performed through combined information coming from different Fe spectral regions, namely Fe2p, Fe3p and valence band (VB). In particular, the latter has been deeply investigated in the literature [38,39,40], therefore special attention was devoted to this part of the spectrum. Representative XP spectra are reported in Figure 3.

Fe2p XP line shape did not change after the electrodecoration, as evident from the overlap of Figure 3a, meaning that no dramatic changes in Fe chemical speciation occurred. Observing the shape of Fe2p spectrum, it resembles more the ones found both for α-/γ –FeOOH and for α-/γ-Fe₂O₃ [40]. Additionally, the curve fitting of this spectral region (Figure 3b) resulted in the typical multiplet associated to mixed iron oxide: four Fe^3+^ peaks (709.0 eV, 710.8 eV, 711.9 eV, and 713.0 eV) and the surface peak (714.0 eV). The “pre-peak” at 708.0 eV mainly associated with Fe^2+^, if any, is very low. Hence, Fe is present predominantly as Fe^3+^, as expected for uncapped nano-systems. Nevertheless, this synthetic strategy was preferred in order to favor a strong core–core interaction between iron oxide and the supported CuNPs.

Much more information was derived from VB regions (Figure 3c,d): that part of a XP spectrum is well-known to be too complex to be fitted, but it appeared to contain features belonging to ferric and ferrous oxide spectra [41,42]. Therefore, from the overall information (O/Fe ratio, Fe2p curve fitting and VB) it was possible to state that our magnetite, both as prepared and copper modified, is indeed in a mixed form of Fe_2_O_3_/FeOOH. However, comparing the VB spectra (Figure 3c) important differences were visible in the region near the Fermi level, where the hybridization of Fe3d states strongly influences the line shape [43]. These findings included deepened overlapping VBs of the bare Fe_3_O_4_, the CuNPs@BDHAC and the Cu@Fe_3_O_4_ (Figure 3d) and determining the valence band maximum (VBM) by linear extrapolation. The energy band gap value (Eg) for CuNPs was found to be 0.8 eV, compatible with the value for copper oxide [44,45], and coincident with the value for bare magnetite. In the latter case, the value has been calculated to be in the range 0–2 eV, depending on the particle size. The most interesting result is the shift to 0.3 eV for the electrodecorated sample, indicating the occurring of alignment of the Fermi levels at the Cu–Fe material interface [44,45].

The higher sampling depth of the VB region in respect to the core level (due to higher kinetic energy values) could be one of the possible reasons why this spectral region has proven to be more sensitive to changes in the surface chemical composition/speciation [41].

All these pieces of evidence are in good agreement with the TEM results, and suggest that a possible interaction between copper and iron oxide cores occurred.

### 2.3. Magnetic Characterization of the Bare Magnetic Particles

For magnetic hyperthermia, the iron oxide nanoparticles should be superparamagnetic at room temperature, and their magnetic interactions should be moderate. For an adequate electrodecoration, we should also avoid particle aggregation. To assess these properties, the SPIONs were magnetically characterized before their electrodecoration.

This characterization is essential in the uncapped SPIONs to check that their magnetic quality is good enough for the application despite the absence of a protective shell. Given that the electrodecoration was successful in the uncapped particles, the magnetic characterization results shown here exclusively regard them. Indeed, the Cu diamagnetism will not significantly affect the magnetic properties relevant in the framework of this study. On the other hand, the magnetic properties of both capped and uncapped particles, before and after Cu addition, will be the matter of further investigation.

**Figure 4:**
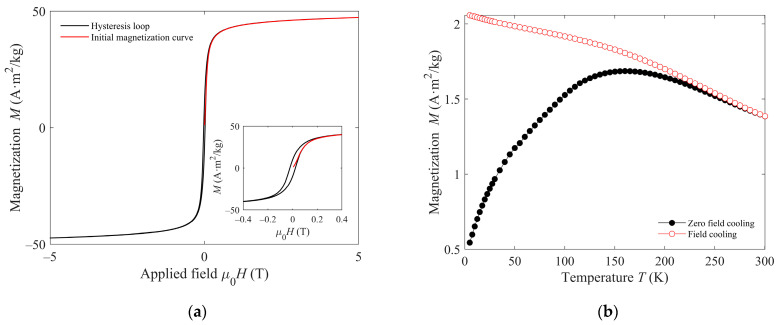
(**a**) Initial magnetization curve (red) and hysteresis loop (black) at 5 K; Inset: detail of the low-field region; (**b**) Zero field cooling–field cooling curves obtained with an applied field of 2 kA/m.

The initial magnetization curve and hysteresis loop at 5 K are shown in Figure 4a. Both curves show quasi-saturation at the maximum applied field (5 T). The law of approach to saturation [46] was used to obtain the value of the saturation magnetization *M_S_* by fitting the experimental initial magnetization data to:

M = M_S_ (−a/H − b/H^2^), (1)

where a and b are free fitting parameters. The fit yields a value of M_S_ = 50 A·m^2^/k. To assess the superparamagnetic character of the particles at room temperature and above, zero-field-cooled (ZFC)/field-cooled (FC) measurements have been performed up to 300 K, which can be seen in Figure 4b. The curves show the characteristics of a blocking process with increasing temperature. Typically, the ZFC curve presents a maximum at a temperature related to the average blocking temperature. Above such temperature, the nanoparticles have a superparamagnetic behavior. The temperature at which the two curves, ZFC and FC, merge is known as the irreversibility temperature, above which even the largest particles in the sample are superparamagnetic. Hence, Figure 4b indicates that all the nanoparticles in the sample show superparamagnetism at room temperature [47].

**Figure 5:**
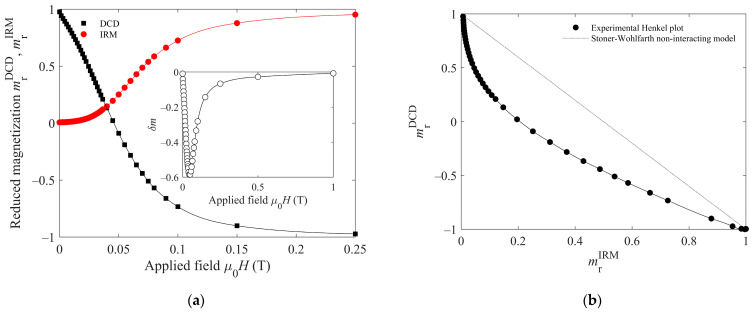
(**a**) DCD (black squares) and IRM (red circles) curves of the superparamagnetic iron oxides (SPIONs); Inset: δm curve. (**b**) Circles: Henkel plot of the SPIONs; the solid line is a line to guide the eye; the dotted line is the reference line corresponding to non-interacting particles.

The moderate decrease in the FC curve in Figure 4b indicates some interaction among the particles, consistent with the absence of capping shell. The remanence plot technique has been used to investigate the nature of such interactions. The technique relies on the difference between the magnetization curves measured by the IRM and DCD methods. The key difference in both curves is that the former starts for each measured point from the demagnetized state, while the latter begins with the sample’s magnetic saturation. Both experimental curves are plotted in Figure 5a, the horizontal axis representing the applied magnetic field H, and the vertical axis the reduced remanent magnetization calculated as the magnetization relative to the saturation value (m_r_ (H) = M_r_ (H)/M_r_ (H→∞)).

In the absence of magnetic interparticle interactions, the reduced IRM magnetization (m_r_^IRM^) and the reduced DCD magnetization (m_r_^DCD^) for the same applied field H should differ, such as m_r_^DCD^ = 1–2 m_r_^IRM^, which would be the reference dotted line in Figure 5b. The experimental data lying below such reference line (Henkel’s plot) are a clear indication of negative interactions [48]. Another way to visualize the interactions is the δm = m_r_^DCD^ − (1 − 2 m_r_^IRM^) curve, presented in the inset of Figure 5a, which, in accordance with Henkel’s plot, shows only negative values of δm. The negative (or demagnetizing) interactions deduced from this method indicate that the dominant effect is the particles’ magnetostatic interplay. This is a consequence of the magnetic field produced by the individual particles at the others’ positions. In contrast, positive dominant interactions would account for magnetic exchange. Exchange interaction is a very short-range one and would only be significant if the crystalline grains would share common boundaries [49], forming aggregates. Then, we conclude that the nanoparticles have some agglomeration but no appreciable aggregation.

### 2.4. Magnetic Hyperthermia of Electrodecorated Particles

SPIONs have the unique advantage of being able to be reversibly activated and deactivated under demand by applying an external magnetic field and without exhibiting persistent remanence or magnetic aggregation, as presented in the ferromagnetic microparticles. Taking advantage of this, different applications are envisaged by different authors, as nanocarriers for drug delivery and magnetic hyperthermia therapies for non-invasive treatment of tumors [16,17,41]. Since only relatively small increases in temperature produce a large effect on cancer cell viability, single domain MNPs can be efficiently used to absorb power from the field through magnetic relaxation processes and dissipate it through a temperature increase in the tumor that triggers the apoptosis the tumoral cells [50].

**Figure 6:**
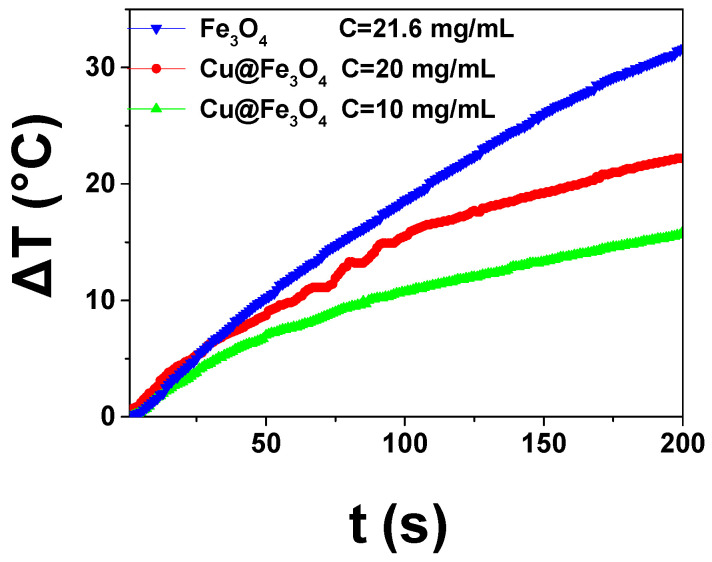
Variation of the medium temperature as a function of the heating time for bare Fe_3_O_4_ particles (blue), and electrodecorated particles at concentrations of 20 mg/mL (red), and 10 mg/mL (green).

Inspired by this, magnetic hyperthermia has been used in this work to heat our hybrid Cu@Fe_3_O_4_ systems, and induce enhanced copper ions release, which has been proven to be an important factor in the modulation of the antimicrobial action [33,35,51]. When using CuNPs, the chemical reactivity is controlled by the surfactant shell formed during their synthesis, so that these substrates can provide an antibacterial action through the release of metal ions. The total amount of the ion release was demonstrated to be directly related to the amount of copper dispersed in the composite material [33,35], while the kinetics is a function of the chain length of the surfactant and so depending on the thickness of the stabilizing shell [52,53]. Considering the property of iron oxide to have superparamagnetic character and antimicrobial action, a further way to tune copper release from Cu@Fe_3_O_4_ by means of magnetic field application has been investigated. A synergistic behavior of copper, BDHAC and iron oxide is therefore envisaged for targeted antimicrobial materials. After assessing the magnetic character of the electrodecorated magnetite, illustrated in Figure 1, a preliminary study was performed to establish the adequate experimental conditions to ensure the copper release by magnetic hyperthermia (we estimated that a temperature around 45 °C should be targeted). To this end, the heating efficiency of Fe_3_O_4_ and Cu@Fe_3_O_4_ NPs dispersions prepared at different concentrations, was measured by magnetic hyperthermia experiments performed under an applied field of 30 mT and 293 kHz. The results are shown in Figure 6 in the form of temperature increases as a function of the heating time for a representative set of samples. They reveal that moderate concentrations of magnetic nanoparticles enable fast temperature increments. A temperature increase close to 30 °C was achieved for Fe_3_O_4_ NPs at a concentration of 21.6 mg/mL after only 3 min of magnetic field exposure. The heating efficiency of electrodecorated Cu@Fe_3_O_4_ NPs dispersions at different concentrations (10–20 mg/mL), shows also intense thermal responses (ΔT = 15–20 °C) in less than 3 min, suitable for under demand applications. It must be highlighted that the thermal performance depends on the concentration of the magnetic NP and also on the composition. It has been reported that Cu doping may enhance the magnetic hyperthermia performance of magnetite [54]; although proving it for our samples is out of the scope of the present work, it should be the focus of a future investigation.

From the temperature increase depicted in Figure 6, the specific absorption rate (SAR), which defines the magnetic power absorbed per mass unit of MNPs, can be computed using the expression:

(2) $$SAR=\frac{m_{NP}C_{NP}+m_{s}C_{s}}{m_{NP}}{\left|\frac{\Delta T}{\Delta t}\right|}_{t_{0}}$$

where *m_NP_*, *m_s_* and *C_NP_*, *C_s_* stand for the mass and specific heat capacity of the magnetic particles and solvent, respectively; then the slope of the temperature rise, ${\left|\frac{\Delta T}{\Delta t}\right|}_{t_{0}}$, is determined from the initial heating (*t*_0_ = 30 s). SAR data compiled in Table 2 were computed taking into account the specific heat capacity of the used solvents, the water (d_H2O_ = 1 g/mL, C_H2O_ = 4.16 J/(g·K)) and acetonitrile (ACN) (d_ACN_ = 0.79 g/mL, C_ACN_ = 2.25 J/(g·K)): tetrahydrofuran (THF) (d_THF_ = 0.89 g/mL, C_THF_ = 1.78 J/(g·K)) mixtures, and magnetite NPs, *C_NP_* = 0.646 J/(g·K), and the mass of each component contained in the measured volume, V = 75 μL. The data reflect that magnetic hyperthermia depends in a non-linear way on a large set of experimental parameters (solvent viscosity and thermodynamic properties, magnetic nanoparticle composition, properties, and concentration) which is still an open question in the field [55]. 

**Figure 7:**
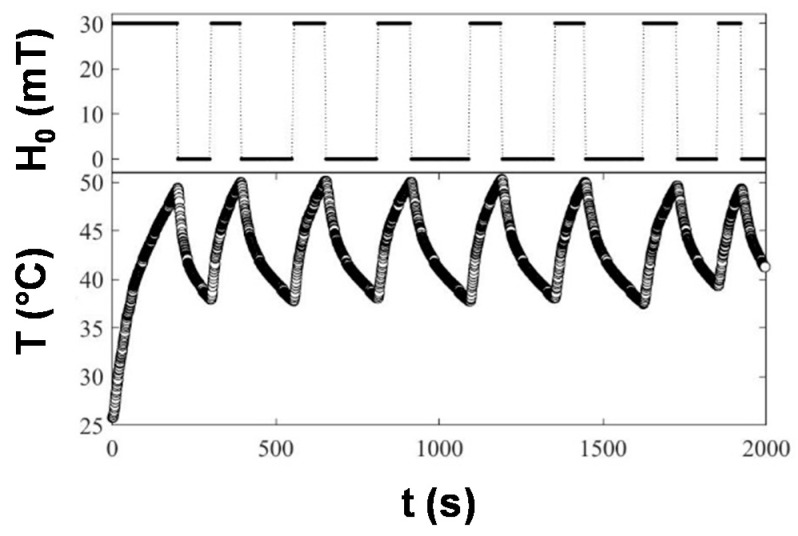
Top: Magnetic field amplitude during the on/off heating cycles for hyperthermia induced Cu release. Bottom: Heating curve (temperature versus time) for a representative sample of Cu@Fe_3_O_4_ MNPs dispersed in a mixture of tetrahydrofuran (THF)/acetonitrile (ACN), with a weight concentration of 20 mg/mL, during 30 min of a magnetic hyperthermia experiment for Cu release.

The results proved that Cu@Fe_3_O_4_ concentrations of 10 and 20 mg/mL were adequate to rapidly achieve the target temperature of 45 °C. We then performed magnetically induced Cu release experiments. With this aim, the particles were exposed to a series of controlled inductive heating cycles to keep it into the desired thermal range of 35 °C ≤ T ≤ 50 °C. In Figure 7, the temperature of a representative sample subjected for 30 min to cycles of switching on/off the magnetic field illustrates how magnetic hyperthermia enables controlled under demand remote thermal application in an easy and precise way.

**Figure 8:**
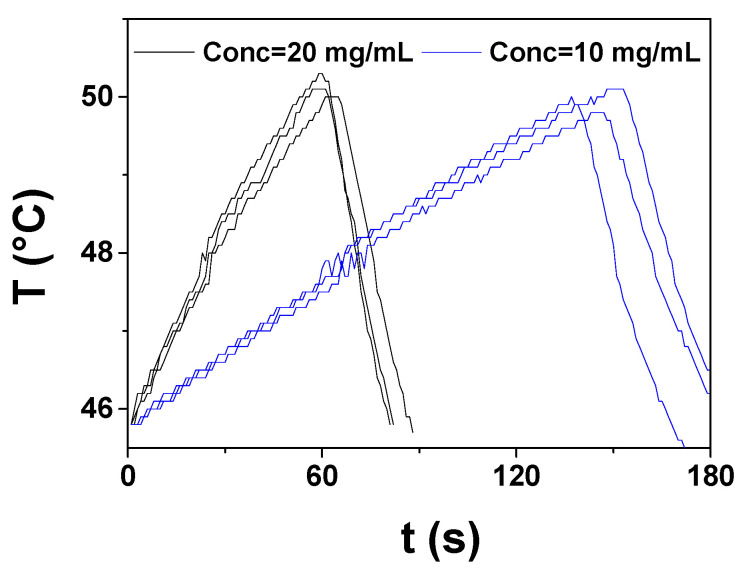
Heating peaks corresponding to two dispersions of Cu@Fe_3_O_4_ MNPs in a mixture of THF/ACN (3:1) with different concentrations (20 mg/mL and 10 mg/mL), subjected to a 30 min treatment of magnetic hyperthermia cycles.

The heating performance can be adjusted by modifying the concentration of the magnetic material or the inductive device parameters (intensity and frequency of the alternating magnetic field) to meet the adequate conditions for each specific application. In Figure 8, heating peak cycles of two dispersions of Cu@Fe_3_O_4_ MNPs with different concentrations (20 mg/mL and 10 mg/mL), subjected to a 30 min treatment of magnetic hyperthermia cycles, have been plotted together to illustrate the effect of magnetite concentration on the heating efficiency. The magnetic nanomaterial concentration allows us to tailor the temperature increase rate (ΔT/Δt), to produce a moderate or fast heating. From the data in Figure 8, we deduce that reducing the concentration of magnetic NPs form 20 mg/mL to 10 mg/mL can lower more than twice the hyperthermia heating rate from 5.72 °C/min to 1.97 °C/min.

The supernatant of the samples was analyzed by ICP-MS to determine the amount of Cu ions after different storing times, in which conventional diffusion of ions occurred, and immediately after the magnetic hyperthermia experiments, to assess the benefits of using inductive heating for an enhanced and sustained copper release. To produce a sharp release of Cu ions C_CuNPs_ = 20 mg/mL was selected, to test the triggering effect of magnetic hyperthermia under sharp conditions. Table 3 reports the copper release amount as a function of the storage time and 30 min magnetic hyperthermia cycle.

From the values reported in Table 3, it is possible to observe the copper release amount over time (3 or 5 days) that naturally occur in the solution. When triggered by magnetic hyperthermia, this amount is multiplied by three. Based on such an experimental evidence, it can be inferred that supporting core-shell CuNPs on superparamagnetic iron oxide is a valid pathway to specifically tailor SPIONs to ensure a controlled release of copper by application of an alternating magnetic field. Along with the copper loading amount and the nature and thickness of the CuNPs stabilizer shell [33,35,53], magnetic hyperthermia proved to be a further and orthogonal way to tune copper ions release, hence the antimicrobial effectiveness modulation can be easily envisaged.

## 3. Materials and Methods

### 3.1. Materials

Iron (II) chloride tetrahydrate (FeCl_2_ ∙ 4H_2_O), iron (III) chloride hexahydrate (FeCl_3_ ∙ 6H_2_O), hydrochloric acid ACS reagent 37%, polyacrylic acid, polyetherimide, benzalkonium chloride, acetonitrile, and tetrahydrofuran were purchased from Sigma-Aldrich (Italy). Copper foils were purchased from Goodfellow. Ammonia solution 30% (NH_4_OH) was supplied by Carlo Erba Reagenti (Italy). None of the reagents were further modified before use.

### 3.2. Methods

Magnetite nanoparticles were prepared by a co-precipitation method [56] of an aqueous solution with Fe^3+^ and Fe^2+^ salts (2:1 ratio, respectively) containing 0.01 M of HCl to prevent any further oxidation of the Fe^2+^. The solution is magnetically stirred while a continuous N_2_ flowing avoids any oxidation during the reaction. An ammonia solution was directly and carefully added to induce the precipitation. The main reaction of the process can be represented as follows:

(3) $$Fe^{2+}+2Fe^{3+}+8OH^{-}\to Fe_{3}O_{4}+H_{2}O$$

For the particles capped with the polyacrylic acid (PAA), a solution in deionized water containing 3.90 g of PAA, while for the polyetherimide (PEI) ones, a 0.01 M HCl solution containing 2.37 g of PEI were prepared. Both solutions were added to the reaction just after the ammonia. Then, the nanoparticles formed, both capped and uncapped, were left to grow for 1 hour at 60 °C under magnetic stirring. After cooling to room temperature, the black solutions obtained were washed with distilled water assisted by magnetic separation up to three times to get rid of the remaining ions. The capped samples were not processed anymore, while for the uncapped one, electrostatic stabilization was achieved by decreasing the pH to 3.

Magnetite nano powders were electrodecorated with copper nanoparticles (CuNPs) using sacrificial anode electrolysis (SAE) [33,35,52]. The electrodecoration was performed in a conventional three electrode cell: the working electrode consisted of a copper plate and the counter electrode was a platinum plate, both with thickness of 0.5 mm and area of 2.5 cm^2^ for an overall solution volume of 5 mL. Reference electrode was a lab-made Ag/AgNO_3_ 0.1 M in acetonitrile. Th electrosynthesis solution contained 20 mg of Fe_3_O_4_ powder and 0.05 M Benzalkonium Chloride (BAC) dissolved in a mixture of acetonitrile (ACN) and tetrahydrofuran (THF) in proportion of 1:3. BAC is commercially available (CAS number 63449-41-2). The applied voltage was +2 V. All the electrochemical experiments were carried out using a potentiostat CH1230B (CH Instrument, Inc, Bee Cave, TX, USA).

Transmission electron microscopy (TEM) analyses of both Fe_3_O_4_ and Cu@Fe_3_O_4_ nanomaterials were conducted using a FEI Tecnai Spirit G2 microscope, equipped with a LaB6 electronic filament gun and operating at a voltage of 120 kV). Samples were diluted 1:100 before placing a drop on a copper grid (TAAB, carbon coated 300 mesh). The magnification factor was 600,000×.

X-ray photoelectron spectroscopy analyses were performed with a Versa Probe II Scanning XPS Microprobe spectrometer (Physical Electronics GmbH, Feldkirchen, Germany), drop casting the colloids on metal slides. The measurements were carried out with a monochromatized AlKα source (x-ray spot 200 μm), at a power of 50.8 W. Wide scans and detailed spectra were acquired in fixed analyzer transmission (FAT) mode with a pass energy of 46.95 eV. An electron gun was used for charge compensation (1.0 V 20.0 μA). All binding energies were referenced to C1s at 284.8 ± 0.1 eV for adventitious hydrocarbon. Data processing were performed using MultiPak software v. 9.9.0.8, 2018.

To evaluate the magnetic hyperthermia effect on the copper ions release, several samples were treated to eliminate the copper from the medium before the treatments (the copper ions are released spontaneously to a certain degree). With this purpose, the magnetic copper nanoparticles were centrifuged at 10,000 rpm for 5 min and separated from the supernatant. The sample pellet was resuspended in a freshly mixture of THF/Acetonitrile 3:1. Then, some samples were left untreated while others were subjected to magnetically driven hyperthermia treatments.

The magnetic hyperthermia was performed using an inductive heating prototype device equipped with a fiber optic thermometer. A fixed volume of (V = 75 µL) of aqueous solution of magnetite electrodecorated by CuNPs (with concentrations of 10 and 20 mg/mL) was placed in the glass sample holder, isolated in a vacuum chamber, generating adiabatic-like conditions. The sample was exposed to the AC magnetic field with fixed amplitude and frequency (30 mT and 293 kHz, respectively) generated by the source while the medium temperature was monitored. 

To assess the potential of magnetic hyperthermia to enhance the Cu ions release, samples with different nanomaterial concentrations were tested. With the aim of keeping the temperature around the target value of 45 °C, the current source was switched on and off cyclically during the experiment. In such a way. the temperatures were between 40 °C and 50 °C for 30 min, as can be seen in Figure 7.

The supernatant was analyzed before and after the hyperthermia treatment to determine the amount of copper released using an inductively coupled plasma-mass spectrometry (ICP-MS) with a NexION 300X ICP-MS system (Perkin-Elmer, Waltham, MA, USA).

DC magnetization measurements were performed using a Magnetic Properties Measurement System MPMS XL-5 (Hmax ± 5T, Quantum Design, San Diego, CA, USA). For this, the colloidal samples were placed inside polycarbonate capsules. Zero-field-cooled (ZFC) and field-cooled (FC) magnetization measurements were carried out by cooling the sample from room temperature to 5 K in zero magnetic field; then, a static magnetic field of 2.5 mT was applied. The ZFC curve was measured during warming up from 5 to 300 K, whereas FC was recorded during the subsequent cooling. The field dependence of the remanent magnetization was measured using the IRM (isothermal remanent magnetization) and DCD (direct current demagnetization) protocols. The initial state for an IRM measurement is a totally demagnetized sample cooled in a zero magnetic field. In the present case, an external field was applied for 10 s, then it was switched off and the remanence was measured (IRM). The process was repeated, increasing the field up to 5 T. In a DCD measurement the initial state is magnetic saturation. An external field of 5 T was applied for 10 s, and then a small external field in the opposite direction to the magnetization was applied; after 10 s, it was switched off and the remanent magnetization (DCD) was measured. This was iterated while increasing the applied field up to 5 T.

## 4. Conclusions

In the present work, the largely known antibacterial properties of copper NPs and their application in material and life science provided the opportunity to work on new nano-systems aimed at exploiting the biochemical and physical properties of materials such as magnetic nanoparticles. A promising field is the one related to magnetic NPs, having recently been studied as nanosystems for cancer therapies by means of magnetic hyperthermia. Benzyl-dimethyl-hexadecyl-ammonium chloride (BDHAC) was tested in previous studies as a stabilizing surfactant of CuNPs to exploit the synergistic antibacterial effect of copper ions and BDHAC. The high affinity of this ammonium salt for the electrodecoration of magnetite was noticed. The aim was to take advantage of the synergistic effect among BDHAC, copper and iron oxide, as they all have an antimicrobial effects.

TEM and XPS experimental evidence resulted in good agreements in understanding the optimal electrochemical parameters for the electrodecoration of Fe_3_O_4_. The final hybrid nanomaterials also proved to preserve the magnetic heating capacity, as evident from the magnetic characterization results.

Finally, ICP-MS experiments proved that copper release from the composite nanomaterials is increased (doubled) when hyperthermia conditions are applied. These promising results pave the way to develop an unprecedented application of the magnetic hyperthermia based on smart composite nanomaterials with antimicrobial properties through a finely tunable ionic release. The latter, in particular, can be controlled by three orthogonal approaches, e.g., by increasing the metal NP loading [33,35], by changing shell thickness [34], and by the application of the magnetic field (this paper), when bioactive NPs are supported on SPIONs.

## Figures and Tables

**Table 1:** X-ray photoelectron spectroscopy surface atomic percentages of Fe_3_O_4_ and Cu@Fe_3_O_4_ samples. Values are expressed as mean values ± 1σ (*n* = 6).

Sample	% Fe	% Cu	% O	%Oox	O/Fe	% C	% N	% Cl
Fe_3_O_4_	22 ± 2	--	55 ± 2	44 ± 1	1.9 ± 0.2	23 ± 3	--	--
Cu@Fe_3_O_4_	11.0 ± 0.7	12 ± 1	35 ± 10	21 ± 3	2.0 ± 0.6	33 ± 4	0.8 ± 0.3	0.5 ± 0.5

**Table 2:** Specific absorption rate (SAR) values of different dispersions of Fe_3_O_4_ and Cu@Fe_3_O_4_ NPs.

Sample	Concentration (mg/L)	SAR (W/g)
Fe_3_O_4_	21.6	41.89
Cu@Fe_3_O_4_	20	16.32
Cu@Fe_3_O_4_	10	27.93

**Table 3:** Copper amount released from two samples of Cu@Fe_3_O_4_ MNPs dispersed in a mixture of THF/ACN at a concentration of 20 mg/mL, as a function of storage time or magnetic hyperthermia (MH) cycles.

**Sample 1**	**Copper Release (mg/L)**
Storage time t = 3 days	425 ± 5
MH t = 30 min	1500 ± 5
**Sample 1**	**Copper Release (mg/L)**
Storage time t = 5 days	780 ± 5
MH t = 30 min	1490 ± 5

## Data Availability

The data presented in this study are available on request from the corresponding author.
